# Food Habits of Older Australians Living Alone in the Australian Capital Territory

**DOI:** 10.3390/geriatrics5030055

**Published:** 2020-09-18

**Authors:** Elizabeth Low, Jane Kellett, Rachel Bacon, Nenad Naumovski

**Affiliations:** 1 Discipline of Nutrition and Dietetics, Faculty of Health, University of Canberra, Bruce ACT 2617, Australia; jane.kellett@canberra.edu.au (J.K.); rachel.bacon@canberra.edu.au (R.B.); nenad.naumovski@canberra.edu.au (N.N.); 2 Functional Foods and Nutrition Research (FFNR) Laboratory, University of Canberra, Bruce ACT 2617, Australia; 3 Department of Nutrition and Dietetics, School of Health Science and Education, Harokopio University, 17676 Athens, Greece

**Keywords:** meal, aged, community dwelling, one-person household

## Abstract

The link between adequate nutrition and quality of life for older persons is well established. With the proportion of older adults increasing, policy regarding support and care for the ageing has shifted emphasis to keeping older adults in their homes for as long as possible. Risk of malnutrition is an issue of importance for this population and, while this risk is well researched within the hospital setting, it is still relatively under-researched within the community-dwelling elderly, particularly with respect to the lived experience. This qualitative study (underpinned by interpretative phenomenology philosophy) explores how the lived experiences of community-dwelling older people living in one-person households in the Australian Capital Territory (ACT) influences dietary patterns, food choices and perceptions about food availability. Using purposeful and snowballing sampling, older people (65 years and over) living alone in the community participated in focus group discussions triangulated with their family/carers. Data were thematically analysed using a previously established approach. Participants (*n* = 22) were interviewed in three focus groups. Three themes were identified: active and meaningful community connectedness; eating well and behaviours to promote dietary resilience. Of these, community connectedness was pivotal in driving food patterns and choices and was a central component influencing behaviours to eating well and maintaining dietary resilience.

## 1. Introduction

Population ageing is a recognised issue in developed countries [1], with the proportion of the population over the age of 65 years increasing. Within Australia, persons aged 65 years and over account for 16% of the population [2], projected to rise to about 18% by 2027 [3] with comparable population for the Australian Capital Territory (ACT) about 13% (55,465 people) [2]. Additionally, a study undertaken by the Australian Institute for Family Studies showed that Australians, particularly older Australians, are increasingly living alone [4]. The findings also identified that nearly 24% of Australians aged between 70–79 years will live alone, increasing to 34% for those aged 80 years and over [4].

The link between adequate nutrition and quality of life in older persons is well established [5,6,7,8]. However, nutritional vulnerability increases with age for a variety of reasons namely, age related physiological changes [9,10,11], psychosocial factors [9,10], reduced mobility [9,10,12] and changes in financial circumstances [8,9,10]. Sustaining nutritional well-being enables maintenance of health and capacity to prevent disease enabling older people to maintain independence and quality of life [11,13].

To better manage the needs of increasing numbers of ageing Australians, current federal government policy (*Living Longer, Living Better*) has shifted emphasis to the provision of support and care to maintain older Australians in their homes for as long as possible [14,15]. While there is a significant body of nutritional research for those aged over 65 years in high-care facilities and hospitals [16,17], there is a paucity of published data, particularly with respect to the lived experience, for those who are living within the community setting [7,12,18] and who are increasingly living alone [4].

The aim of this study was to explore how the lived experiences of community-dwelling older people living in one-person households in the ACT influence dietary patterns, food choices and perceptions about food availability. Through use of a qualitative approach, this unique study provides insights for issues based on the lived experience for a group of older Australians aged 65 years and over, and for their family/carers.

## 2. Materials and Methods 

### 2.1. Study Design

This was a qualitative study underpinned by a philosophy of interpretative phenomenology. This philosophy was chosen so that the data (stories/responses) from participants could be analysed in a way that uncovered the underlying meaning(s) in the data—that is, moving beyond the descriptive [19,20]. Data were collected through the use of focus groups. Descriptive statistics were recorded to contextualise and describe primary participants only. A mini mental state examination (MMSE) [21] was administered as a screening tool to ensure that participants were cognitively capable. A mini nutritional assessment short form (MNA-SF) [16,18] was administered by two researchers (J.K. and E.L.) just prior to focus groups. The MNA-SF was used to screen primary participants to identify if any were at risk of malnutrition, and to provide advice back to these participants if their MNA-SF score indicated risk of malnutrition. Secondary participants were not screened for cognition (MMSE) or malnutrition (MNA-SF).

The University of Canberra Human Ethics Committee provided ethics approval for this study (HREC 16–38), and this study was performed in accordance with the Declaration of Helsinki [22] Informed written consent was obtained from all participants prior to commencement of the study.

### 2.2. Population

#### 2.2.1. Sampling Process

Two groups of participants for focus groups were sought—primary participants, those aged 65 years and over, living alone and independently in the community; and their family/carers (secondary participants). By focusing on older community-dwelling participants living alone, the influence of other person(s) within the household on pattern of consumption and food choice was removed [23]. Sampling used purposeful and snowballing techniques through advertisement over four weeks in the weekly pew bulletin of a large, mainstream Christian church (the church) within the ACT. Members of the church are not required to observe any religious dietary restrictions and come from a diverse range of groups within the ACT community.

Family/carers of primary participants were approached separately, and with the knowledge of the primary participants. The family/carer group was included to enable triangulation of responses and exploration of the alignment of the perceptions of the primary participants with those of family/carers.

#### 2.2.2. Inclusion Criteria

Primary participants were required to be aged 65 years or older, and living alone and independently in the community. A mini mental state examination was administered for all primary participants with a MMSE score of at least 25 required [21]. The MMSE was administered to ensure cognitive capacity of primary participants [21]. Additionally, primary participants needed to be able to attend one of the two scheduled focus group times.

Secondary participants were family members or carers with a role as a carer for a primary participant.

#### 2.2.3. Data Collection

Focus groups, lasting 55–90 min, were conducted in a community hall in the ACT and were facilitated by the team of researchers (E.L., J.K., R.B.). Semi-structured interview questions were used, and the same questions were asked at all focus groups (Supplementary Materials Table S1). Questions were developed in consultation with academic experts in this area of research. Audio recordings of the sessions were transcribed verbatim. Transcripts were inductively analysed and manually coded (E.L.) using an approach previously established by Braun and Clarke [24]. This is a six-phase approach starting with the researcher transcribing the data and then iteratively searching the data to identify subthemes that are then grouped by themes (as used in several previous studies) [25,26,27]. It also has to be emphasised that successful focus groups require genuine interactions between the facilitator and participants [20]. The potential for the perspective of the author to influence the focus group through participation is acknowledged. Reflexivity was applied during the coding process [28]. Additionally, themes and transcripts were crosschecked by two coauthors (J.K. and R.B.).

## 3. Results

Three focus groups using semistructured interview questions were undertaken. Two focus groups (Group 1 and Group 2) involved primary participants (*n* = 9, *n* = 7) and one focus group (Group 3) involved family/carers (*n* = 6). Primary participants (*n* = 16) ranged in age from 65 to 93 years (median age 84.2 years) with 13 women and three men participating. The primary participants attended one of two focus groups offered. For primary participants, choice of focus group, that is Group 1 or Group 2, was determined by the primary participant and dependent on convenience for them. No participant who was able to attend the scheduled focus groups was deemed ineligible to participate. More members of the church did express interest in participating in this study but were unable to attend the scheduled focus groups. These potential volunteers were excluded.

Participants in Group 1 (*n* = 9) were all women. Group 2 (*n* = 7) comprised three men and four women. Group 3 (*n* = 6) were family members (one man and five women) who were primary carers for five primary participants. One family/carer participant was also a primary participant representing an emerging scenario of older people caring for parents and provided insights from a dual perspective.

All primary participants were free-living within the community, residing in their own homes or in independent-living homes within retirement villages. Most participants were known to each other. No evidence of reticence to speak or voice opinion was observed during focus groups. All but two primary participants were long-term and active members of the church. The remaining two primary participants had a tangential association with the church. 

During the focus groups, primary participants disclosed a range of health and mobility challenges namely, use of walkers, vision and hearing impairment and medical conditions. Some primary participants mentioned the impact of medications on timing of food consumption but did not disclose more specific information. With the exception of two primary participants, all primary participants had MNA-SF scores indicating no risk of malnutrition; that is, scores greater than 12. While two primary participants had MNA-SF scores flagging risk of malnutrition (scores in the range 8–11), they advised that they were already receiving appropriate medical support.

Responses to focus group questions were consistent across all groups except in one area relating to support. Primary participants underestimated (though not undervalued) the amount of support provided by family. Due to the consistency of responses, the authors agreed that there was no requirement to undertake additional focus groups. Responses between the family/carers group matched those from their family member, enabling triangulation of responses across groups. Three themes emerged from the analysis of transcripts: (1) active and meaningful community connectedness; (2) eating well and (3) engaging in behaviours to positively overcome challenges to eating well. Key ideas underpinning the themes and sub-themes are summarised at Table 1.

### 3.1. Theme 1—Community Connectedness

This theme encompasses the role of active and meaningful engagement with one or more communities in supporting healthy eating patterns, sound food choices; and motivating adaptive strategies should challenges arise:

“I think you need something outside otherwise you’d just melt away in your own house. You’d go downhill pretty quickly if you didn’t have something.”

G1, primary participant.

Participants acknowledged the importance of community connectedness and signalled that this provided a sense of purpose and a pathway for participation and meaningful contribution. This in turn provided motivation to eat well and remain healthy as well as means to living positively:

“I’ve got to live and be independent and get out and do things and feel I’m making a useful contribution and that helps me to come home and live, otherwise I’d get depressed.”

T1, primary participant.

Primary participants’ responses demonstrated that being active within a community held a variety of meanings including providing a network of support outside of paid carers and family; opportunities to maintain a range of interests; keep fit; and generated social outings.

*“It’s the gathering more than anything—it’s the talk to other people.”*

J1, primary participant.

Responses from primary participants and family/carers suggested that activities that were meaningful may have gender-specific tendencies with women in this organisation participating in activities relating to service and the provision of food:

“Yeah she’s gone to making simple things … Yeah cause she feels that … she still needs to give something. And a couple of things she belongs to she takes food to those for afternoon teas and stuff.”

L1, family/carer.

Responses from male primary participants and their family/carer indicated that men were more focused on activities such as volunteering, or connections with sporting organisations.

*“I go to a retirement village to read poetry regularly…”*

T1, primary participant.

*“…well he’s with the (organisation) so he goes out a couple of nights a month. … Saturdays—we’ll take him to a game of football. He’ll watch, you know, son-in-laws, grandson-in-laws, grandsons playing and things like this.”*

A1, family/carer.

### 3.2. Theme 2—Eating Well

This theme was more complex than eating healthy foods and covered a number of areas that impacted food choices, eating patterns and perception of food availability.

*“I think what I eat is balanced—balanced diet. And a lot of it is what I grew up with.”*

M1, primary participant.

Eating patterns and food choice were generally uniform across the two primary participant groups and heavily influenced by habituation. Participants followed meal patterns and food choices that were ingrained from childhood and early adulthood. These were also formative in the development of perceptions regarding healthy eating and food preparation. There were numerous references relating to what the participants ate as children, with a notable emphasis on eating vegetables:

The meal pattern was breakfast, lunch and dinner but always guided by hunger—similarly for snacking. Deviations were driven by either a change to daily routine (temporary) or medical issues. For example, type 2 diabetes mellitus and moving to smaller more frequent meals to better manage blood sugar levels. Despite the habituation, all participants were open to trying new foods with a number of participants sharing their experiences.

*“I have things I like to eat and work out a pattern of it. I don’t stray from it very often.”*

B1, primary participant.

*“Dad’s very regimented in what times he eats.”*

A1, Family/carer.

Food choice was influenced by access. No primary participant advised difficulty in accessing foods, though meat prices and seasonal availability of fresh produce (and associated pricing) were cited as reasons for moderating choice. Primary participants ensured that they always had something for emergencies:

“I never let the pantry or fridge run down to nothing.”

K1, primary participant.

Access issues voiced, related primarily to accessing food in usable quantities from supermarkets.

*“… some of the quantities of food in the supermarket like we were talking before. They are just too big for single people.”*

L1, family/carer.

Additionally, primary participants felt that they were financially penalised for purchasing small quantities, particularly fresh produce and meat. As the primary participants all had preferences for food prepared from scratch and using fresh ingredients, this was a substantial issue for them:

“… (Supermarket chain) have a policy of making it cheaper for large families which makes it terrible for people living alone.”

T1, primary participant.

Other supermarket access issues were availability of Australian produce; constant shifting of products impacting those with vision and mobility issues and changes to product formulation that impacted taste and dentition.

### 3.3. Theme 3—Behaviours to Positively Overcome Challenges to Eating Well

This theme encompasses adaptive strategies implemented to maintain usual food habits and food choices when challenges were encountered, including changing tastes and decreasing mobility. Additionally, these were influenced by a number of other factors including accessing government programs/benefits and asking for support outside of family:

“What concerns me is that too many people after they retire don’t think “this is a new life—what can I do with it? If you don’t have this attitude it reflects on how you live at home. You stop looking after yourself and therefore don’t eat properly.”

T1, primary participant.

Willingness to implement adaptive behaviours and strategies to maintain dietary resilience was evidenced by the substitution of foods that no longer tasted right, use of frozen or pre-cut vegetables, cooking in bulk and freezing to reduce meal preparation time and use of gadgets such as soup machines, pressure cookers or shifting usual eating patterns to facilitate medication requirements:

“Food sometimes (doesn’t) taste right anymore. So, you move on to something else that tastes nicer.”

K1, participant.

Accessing government programs/benefits was a significant issue. Family/carers responses noted difficulties accessing government support and services citing the need to continually advocate on behalf of their parent and actively engage with government departments and providers to receive payments and services:

“We’re having problems trying to get (parent) onto a pension. So (they’re) not on the pension yet. So, until (parent) gets on the pension (they) can’t be assessed for anything else—(they) can’t access services—you can’t get any help.”

K2, family/carer.

Primary participants negatively associated seeking support from outside of their family with loss of independence and diminishing capability. This was the one area where primary participants and family/carers had some differences in responses with primary participants, potentially underestimating the amount of support provided by family/carers who also juggle work and their families’ needs.

*“That would be a step that (parent’s) not prepared to cross yet. Meals on Wheels or frozen meals.”*

G2, family/carer.

Of the three themes, community connectedness was the most strongly expressed, driving primary participants to maintain healthy eating patterns and food choices. Community connectedness acted as a catalyst driving the individual themes of eat well, behaviours to promote dietary resilience and sustaining interaction between them. However, connection with community had to be meaningful and purposeful. It needed to be a two-way process, that is, involve giving as well as receiving. For this group, community connectedness provided support beyond family and multiple opportunities for commensality through participation in group activities. Active participation prompted primary participants to maintain a positive attitude to implementing adaptive strategies to maintain eating well.

## 4. Discussion

The relationship between the three themes used in this research project was exceptionally complex but, nevertheless, consistent with other studies that have found that the factors influencing eating patterns, food choice and dietary resilience are varied, complex and often interrelated [13,23,29]. The importance of community connectedness for the primary participants was consistent with other studies with respect to the importance of maintaining social identity and having a positive impact on nutrition and health through facilitating cultural involvement and social participation [29,30,31].

The findings of this study also identified a potential link between gender and type of activity that provided meaning. For female primary participants, meaningful activities tended to centre on food activities including organisational catering and serving morning tea and lunches. For males, activities were connected with different roles such as providing readings at retirement villages, watching football with grandchildren and socialising with others. While this suggests that gender influences activities that provide meaning and value to participants, due to there being a smaller number of male participants in the study these findings may reflect a gender bias (female bias) potentially influencing the finding. However, there are a number of studies supporting that for women, the provision of meals and food holds different meanings compared with those held by men, including maintenance of personal and social identity [23,32,33,34,35]. For the women primary participants in the study, this was particularly strong with respect to their connectedness with their church community. Provision of food and food related services provided a path for activities and interactions that were both meaningful and purposeful, and provided an opportunity to reaffirm social identity and demonstrate capability.

No gender difference was noted with respect to cooking skills with all primary participants cooking meals from scratch. A study undertaken by Bjørner et al. (2018) looking at meal-time practices among community-dwelling older adults in Denmark also found that male participants had good or very good cooking skills [5]. The findings in this study are likely to be influenced by the small number of male participants and are in contrast to findings from other studies which found males living alone, particularly as widowers, less inclined to be skilled or motivated to cook healthy meals [29,32,33]. Influencing this finding was the high importance primary participants (both men and women) placed on cooking meals from scratch. This was additionally evidenced by the number of participants who indicated engaging in adaptive behaviours to overcome changes to established dietary patterns to maintain a focus on food intake that was consistent with their perception of healthy eating and cooking.

Habituation, coupled with motivation to undertake adaptive behaviours to sustain habituation, were important factors in ensuring that primary participants maintained healthy meal patterns and healthy food choices. That is, for these participants they were important components for establishing and sustaining dietary resilience. This is consistent with the literature [9,13,30,34]. However, primary participants were willing to try new foods. For most, this was in the context of eating out with others and was important when usual foods were no longer tasty or were no longer tolerated, highlighting the role of maintaining connections with community in accessing new foods and promoting adaptive behaviours to maintain dietary resilience. Not wasting food was an important consideration, and eating out additionally provided scope for trying something new without primary participants becoming overly concerned about wasting food if the new food was disliked. Further study of the role of habituation, as both an enabler and barrier in health messaging, should be considered.

As mentioned previously, the interactions between themes was complex, with community connectedness appearing to drive and sustain interactions between and across themes. One of the key factors for this was the facilitation of opportunities for commensality. The role of commensality in maintaining nutritional status and influencing food habits is supported by the literature [36,37,38,39,40]. For example, there is evidence that food intake cues are influenced, and can result in more food being eaten, when eating with others [39], whereas lack of commensality can contribute to loss of appetite [36,40] and provides an avenue for social support [38].

Data collected as part of the malnutrition risk screen evidenced that all but two of the primary participants were well-nourished and not at risk of malnutrition. It is not unreasonable to acknowledge the contributing role of commensality in this. All primary participants were active members of a number of communities both within, and external to, their church community—for example, sporting organisations, bridge clubs and walking clubs. Primary participants noted and valued opportunities for commensality. There were, however, varied underlying motivators for engaging in these opportunities, and these crossed all three themes. For some primary participants it was about the company and not the food (community connectedness), for others it was a way to have a satisfying meal without having to cook for themselves (eating well), and also a way to enjoy a meal that was different to usual food repertoire (eating well and adaptive behaviours).

Access to external government-funded support services is dependent on assessed level of need. Most primary participants negatively perceived utilising this external support as signalling a change to their social identity and as an admission of incapacity and diminishing independence. In turn, this placed greater reliance on family to provide support. Families did not resent needing to provide additional support, but the reticence to seek outside help may limit capacity to build and maintain behaviours that promote dietary resilience, and may result in increased risk of nutritional vulnerability [29]. In turn, this may reduce capacity to maintain community connectedness, impacting access to social support and opportunities to demonstrate value as a contributing member of their community.

Additionally, responses from family/carers noted issues with obtaining services that would support their parent to age well in place, including services that relate to nutrition support such as shopping and food preparation. Issues included inflexible government systems, information silos, and difficulties navigating administrative processes. Some family/carers also noted the current emphasis on assessment of need based on negatively phrased criteria which focus on functional/physiological deficit and, in turn, signals a message of declining capability rather than a message of support. While results from this study cannot be extrapolated across the larger population, this finding suggests that there is risk that accessing appropriate and necessary support to age well in place may be too difficult for a number of older Australians who live alone and are not well supported by family or community.

### 4.1. Strengths

The strength of this study is its uniqueness in looking at food patterns, food choices and perceptions around food access through the prism of the lived experience of the participants, with the added dimension of triangulation of these responses with family/carers [20]. Responses across groups showed consistency across all themes (with one slight deviation with respect to the support subtheme in the theme adapt). This study contributes to the paucity of data on older adults living alone in the community-dwelling setting.

### 4.2. Limitations

Limitations of the study include the sample size and gender balance, and that all participants were connected to a religious organisation indicating a potential spiritual dimension to their responses. Study participation was voluntary with participants primarily being individuals who have an active interest in nutrition, with findings reflecting this interest. One participant belonged to both primary participant and family/carer groups, so may have had opportunity to consider the questions more fully between group sessions. Not all primary participants had family/carers to provide insights, and this may have reduced the strength of triangulation of responses. The study was also case-specific, restricting findings to this study and limiting generalisations to the broader community, although findings did support other research findings. Additionally, there were no follow up interviews that could have enabled greater insight and exploration of comments during focus groups.

### 4.3. Future Directions

Australian government policy focuses on maintaining older Australians within their own homes for as long as possible [14,15]. Successful implementation of this policy with respect to enabling access to services to support older Australians to maintain meaningful and active community connectedness will require both adequate funding and an appropriately trained workforce. There are additional areas impacting community connectedness where this research could be extended. These include differing socioeconomic influences (such as education level, financial and housing status) and different groups of older Australians, identifying those who are not well connected with community to determine if factors such as ethnicity, rural/remote location and access to services/supports impact this theme. Additionally, the role of spirituality in maintaining sense of place in a community could be further investigated, with some focus on a comparison between those who are active members of religious organisations/communities and those more secularly aligned.

The study also suggests that gender may influence what constitutes a meaningful activity. However, there was a bias in gender balance of the sample. Further research could investigate more closely the role of gender and what constitutes meaningful activities, and how these impact on food choices and eating patterns. However, elements of this suggested bias may, additionally, be driven by norms of behaviour based on religious teachings. It would be informative to compare this with those whose behaviours are driven by more secular norms.

One area for consideration for further study relates to the impact of the dual role of older Australians acting both as older Australians and as carers on nutritional status and needs. As the number of Australians ageing increases [2], both parents and adult children (over the age of 65 years) will be impacted. It is not unreasonable to suggest that if adult children become nutritionally vulnerable, this will have a flow-on effect to the parent/s for which they are carers. Further research should be undertaken to explore the impact of negative perceptions towards external support as a barrier to accessing services.

Access to food in supermarkets in single person quantities, and without paying a premium, was identified as an issue by both primary participants and family/carers. For primary participants, preparation of food from scratch was important. As this necessitates purchase of food items from supermarkets, barriers to successfully accessing fresh food and groceries have an immediate impact. The impact of supermarket practices and pricing on food access for older Australians who live alone should be considered for further study.

## 5. Conclusions

This study provides a unique insight through the prism of lived experiences into influences of dietary patterns, food choices and perceptions about food availability for some older Australians. For this group, having a meaningful purpose in life, continuing to be included in community life and having the opportunity to make valued and meaningful contributions to community were important factors for primary participants in maintaining dignity, independence and motivation for ageing actively and positively. Additionally, for some primary participants, being limited to social participation within a community was not enough. This may have future implications for establishing vibrant and positive independent-living aged care communities.

## Figures and Tables

**Table 1:** Summary of key ideas underpinning themes and sub-themes.

**Theme 1: Community Connectedness:** *“I think you need something outside otherwise you’d just melt away in your own house. You’d go downhill pretty quickly if you didn’t have something”.* G1, primary participant.	
**Subtheme**	**Key Ideas**
Valued	Evidenced by responses relating to being included and asked to contribute. “The (organization) is very important and meaningful for her—being here and having that connection.” K2, family/carer.
Motivation	Responses from all participants supported assertion of strong sense of purpose and commitment, a reason/driver to get up every day. Strong sense of personal responsibility for eating well to keep healthy: “If I don’t look after myself, nobody else will.” T1, primary participant.
Social support	Friendships and connections formed through membership of organization and groups within the organization and within larger community: “It’s the gathering more than anything—it’s the talk to other people.” J1, primary participant. Includes sharing information—for example, during focus groups participants exchanged information about a new public transport service for seniors and community transport services. Interestingly, this information coming from peers was received well and seen as a mechanism for positively supporting independence rather than as a sign of decreasing capability impacting independence. Removes fear of social isolation: “My family lives in (city) and I don’t have relations here. But fortunately I live in a cul-de-sac street and the people around me have formed a family and I’m the sort of street grandfather—which pleases me.” T1 primary participant.
Contribute	Contribution had to be meaningful. Responses from participants evidenced that there was a need to give back—not always receiving. “See my Mum would always want to bring something, you know, because that’s part of her nature giving and sharing.” K2 family/carer. Also satisfies a need and provides tangible evidence of still being able and independent. Additionally, this supports feelings of being valued and signals meaningful participation. “She’s gone to making simple things …she still needs to give something.” L1, family carer.
Participation	Responses from participants acknowledged that volunteering, too, was important—generates feeling of inclusion (evidence that tasks modified to ensure older members can participate); provides signal to others in the community of still being able and independent. “… they want to serve morning tea.” K1, family/carer. “... you’ve got to include them … even making stuff because they’ve always made it.” P1, family/carer.
Social	Primary participants advised that they ate out regularly with others within the organization and with those from other organizations. Demonstrates the role of community connectedness in being a driver for generating social occasions. This strengthens feelings of community connectedness as well as presenting opportunities for commensality. “It’s the gathering more than anything—it’s the talk to other people.” J1, primary participant.
Access	More complex than just immediate access to foods. Irritants (though not barriers) identified included supermarkets charging more for smaller quantities, not buying product because of waste generated from larger quantity, lack of Australian produce and frequent changes to supermarket layout. Primary participants maintained adequate food stores for emergencies: “I never let the pantry or fridge run down to nothing.” K1, primary participant.
Physiology	Primary participants’ responses noted impact of ageing on their body—early and prolonged satiety, changes to taste, reduced mobility, impact of medical issues—arthritis, diabetes, diverticulitis, cardiovascular issues, dysphagia were specifically mentioned—food intolerances (fatty, rich foods specifically). This was echoed by family/carers responses and evidenced this is one of several drivers for food choices and meal patterns.
**Theme 2: Eating Well** *“Well—sometimes you don’t feel like eating. You make something and put it down and I get about half-way through it and I think “that’s enough”. So, I don’t eat as much as I used to, but I still enjoy eating.”* SA, primary participant.	
**Subtheme**	**Key Ideas**
Taste	Taste was mentioned frequently—separated as a subtheme from physiology due to impact of food choice. Responses indicated shop bought foods can be too salty— “I steer clear of the packed things because I think they are a bit over-salted.” M1, primary participant. “I’ve learnt not to have pies, sausage rolls (from shops/bakeries). They’ve all got too much salt in them.” K1, primary participant. Primary participants noted an increasing preference for sweeter tasting foods. Taste is a driver for preparation of food from scratch rather than use of preprepared meals. Strong influence on eating well to maintain health: “I think my basic essential diet is really based around what I consider is good food to eat.” J2 primary participant.
Habituation	Food choices, eating patterns and food preparation largely tied to childhood with responses frequently including— *“how you were brought up”*, *“what you grew up with”*; and *“I have things I like to eat and work out a pattern of it. I don’t stray from it very often.”* B1, primary participant.
Flexibility	Despite strong influence of habituation, primary participants were open to try new foods with several discussions occurring between primary participants about experiences. Eating out seen as a good opportunity to do this.
Resilience	Responses demonstrated a motivation to actively implement adaptive eating/food choice strategies in response to barriers—for example, move to smaller more frequent meals in response to onset of diabetes, increasing mobility issues (including increasing impact of arthritis). Motivation an important factor in continuing to eat well with community connectedness an important driver in maintaining dietary resilience.
Convenience	Various strategies implemented to maintain habituated and preferred food choices at home. Use of gadgets common—soup machine, slow cooker, pressure cooker. Foods prepared in bulk and portions frozen to reduce the need to prepare food every day. Primary participants noted the importance of frozen vegetables important as they reduced standing time and preparation and waste (just use what you need).
**Theme 3: Behaviours to Positively Overcome Challenges to Eating Well** *“What concerns me is that too many people after they retire don’t think “this is a new life—what can I do with it? If you don’t have this attitude it reflects on how you live at home. You stop looking after yourself and therefore don’t eat properly.”* T1, primary participant.	
**Subtheme**	**Key Ideas**
Adaptive strategies to overcome challenges to eating well	This subtheme was also linked with *Theme 2: Eating well, subtheme Convenience*. Participants developed, and regularly used, new strategies to maintain preferred food choices and preparation methods. These largely focused on ways to reduce effort or time for food preparation—for example, use of soup makers and slow cookers, cooking in bulk and freezing, use of frozen meals and vegetables, use of prechopped fresh foods and tinned goods (particularly soups). These adaptive behaviours also demonstrated not only a desire to be seen as independent and capable of looking after themselves, but also to maintain habituated preferences in foods and food preparation.
Support	Support from family was valued and preferred to external support. Desire to remain independence may also moderate use of external support services, as this may be perceived as a sign of loss of independence and capability: “… but then again, this is someone (refers to 80 years old mother) who refused to get a senior’s card because she didn’t consider herself old.” K2, family carer. Support from family was preferred to externally sourced support: “When they did Mum’s (aged care assessment) I said one thing you’ve got to tell them is you need your sheets changed. …(she said) You can do that. I said I can do that, but I don’t do it when you want.” L1, family carer. Several family/carer participants echoed this response.
Independence	Some primary participants had a need to not burden family. All primary participants had a determination to remain independent, or at least maintain an appearance of being independent: “(discussing living in an independent living retirement village) … I’m looked after and have emergency call ups and things like that. So, I feel it frees up my children because I’m in a place where I am pretty well looked—I’m pretty independent.” B1, primary participant. Family/carers respect and value the need for their parent to remain independent: “When she had (medical issue) then we actually turned up every day and we cooked. And that’s a balancing act between—we could do it every night but we don’t because I think it’s good for her to have independence.” G2, family/carer. Other participants valued independence, not only as evidence that they could still contribute and be valued members of society, but as a way to maintain good mental health. “I’ve got to live and be independent and get out and do things and feel I’m making a useful contribution and that helps me come home and live otherwise I’d get depressed.” T1, primary participant.
Government	This was both an enabler and barrier to government funded support services, including assistance with shopping and meal preparation/provision. Navigation of administrative requirements and processes can be difficult. Family/carers spoke of difficulties and ongoing need to advocate for parent to gain/retain access to services: “The amount of hassle I had to get Meals on Wheels for (parent) with the government and their bright idea of MyGov because that part wasn’t talking to (different government department). It took eight weeks to even get to go and pick a meal so (parent) could go and have a look to see if he would like to try Meals on Wheels. It took eight weeks.” A1, family/carer. Family/carer participants also noted that access requirements for government support are based on need (limitations) of applicant and raised concerns about negative tone of assessment interviews. That is the interview/assessment focuses on what the applicant can’t do, raising concerns that this potentially causes negative perceptions about the process by primary participants by identifying incapacity and loss of independence rather than as a means to maintain independence: “But it’s that independence. …I imagine her age group (90 years plus) there could be people who need more help or entitled to more help than they are getting but don’t want it because it’s a decision—you’ve got to say you’re less independent.” G2, family/carer.

## Supplementary Materials

The following are available online at https://www.mdpi.com/2308-3417/5/3/55/s1, Table S1: Focus group interview questions.
