# Supplementary material for: Food Habits of Older Australians Living Alone in the Australian Capital Territory
Source: Geriatrics (Basel). 2020 Sep 18;5(3):55. doi: 10.3390/geriatrics5030055 (PMC7555807; doi:10.3390/geriatrics5030055)
Supplement: Supplementary file 1 [file geriatrics-05-00055-s001.pdf]

**Supplementary Material**

**Table S1.** Focus group interview questions.

| Questions | Prompts (if required) |
|---|---|
| ***1. Food patterns:***<br>*Typical Day*<br>*Primary participants:* Tell me about what and when you eat on a typical day.<br><br>*Family/carers:* Tell me about what and when your family member eats on a typical day. | How often do you/they eat alone?<br>What things drive the times that you/they eat? (for example, appointment times, medications, carer availability to assist with food preparation, use of Meals on Wheels, eating out.)<br>Do you/they prepare food by themselves/yourself or do you have assistance?<br>Do you/they buy much pre-prepared/ready to eat food (not fast food)?<br>What does it take for a day to become different?<br>How often would this happen? |
| *Atypical Day*<br>*Primary participants:* Tell me about these days and how these days make your eating patterns different.<br><br>*Family/carers:* Tell me about these days and how they alter your family member's eating patterns.<br>*If required—Eating out:*<br>Tell me about the impact of eating out on your/their eating patterns for the day? | Does this happen often/regularly?<br>Is it planned or unplanned?<br>Do you/they enjoy this experience?<br>Do you think that you/they eat more or less when you/they eat out—why? |
| ***2. Eating and food choices:***<br>Tell me about the sorts of foods that you/they like to eat?<br>Can you tell me a bit about what it is about these foods that causes you/them to like them? | Why do you/they like what you eat? |
| **3. Importance of community**<br>*Primary participants:* How important is community to you? | If required, be more explicit about community meaning personal interest groups, neighbors. |

| Questions | Prompts (if required) |
| --- | --- |
| *Family/carers:* How important is community to your family member? | |
| ***4. Perceived food availability:***<br>*Preamble:* The final area we are going to look at is food availability. This area may cause some discomfort so please keep in mind that participation is voluntary. Additionally, if you do feel uncomfortable with the discussion, I am happy to hear your view privately at the end of the session.<br><br>Food availability is impacted by a number of factors—affordability, access to shops (mobility issues), capacity to prepare food, access to foods you can eat (particularly if you/they are on a special diet for conditions such as high cholesterol or diabetes).<br><br>*Primary participants:* I'd like to hear about times you have experienced difficulties in accessing food or been concerned that you may not have been able to access food.<br><br>*Family/carers:* I'd like to hear about times you think that your family member have experienced difficulties in accessing food or you have been concerned that they may not have been able to access food. | |
